# Prenatal diagnosis of rare genetic disorders: fourteen years’ experience of a tertiary genetic centre from India

**DOI:** 10.1186/s13023-025-04003-9

**Published:** 2025-09-02

**Authors:** Jayesh Sheth, Tejasvi Dhondekar, Manali Ajagekar, Chaitanya Datar, Archana Kher, Jigish Trivedi, Swati Thakkar, Ajit Gandhi, Meenakshi Soni, Mayank Chaudhary, Manish Banker, Anil Jalan, Mamta Muranjan, Sujal Munshi, Ami Munshi, Mili Pandya, Jhanvi Shah, Aadhira Nair, Riddhi Bhavsar, Frenny Sheth, Harsh Sheth

**Affiliations:** 1 FRIGE’s Institute of Human Genetics, FRIGE House, Jodhpur Gam Road, Satellite, Ahmedabad, 380015 India; 2 Bharati Hospital and Research Centre, Dhankawadi, Pune, India; 3 Seven Hill’s Hospital, Marol Maroshi Road, Andheri (E), Mumbai, India; 4 Pulse Hospital, Ambavadi, Ahmedabad, India; 5 Sonography & Color Doppler Clinic, Dharnidhar Cross Road, Vasna, Ahmedabad, India; 6 Unique Hospital, Solapur, India; 7 Shivam Hospital, 4-Mahaveer Colony-B, Bhaskar Choraha, Jodhpur, Rajasthan India; 8 May flower Hospital, Memnagar, Ahmedabad, India; 9 Nova IVI Fertility, The Pulse Woman Hospital Pvt. Ltd., Navarangpura, Ahmedabad, India; 10 Nirman Metabolic Clinic, Akshar Business Park, Vashi, Navi Mumbai, India; 11 Department of Pediatrics, KEM Hospital, Parel, Mumbai, India; 12 Kalpana Munshi Hospital, Narayan Nagar, Ahmedabad, India

**Keywords:** Rare disease, Diagnosis, Treatment challenges, Prevention, Prenatal diagnosis, Awareness, Affordable

## Abstract

**Background:**

Rare genetic disorders are increasingly diagnosed due to advancing genetic technology, whilst, treatment for them is challenging. Therefore, their prevention by prenatal diagnosis is a way forward to reduce the overall burden. The present study provides an overview of a cohort of patients who were offered prenatal diagnosis for genetic disorders at a tertiary genetic center in India.

**Methods:**

The study included 1,738 prenatal samples for the period of 2008 to 2022, identified as being at high risk for rare genetic disorders based on family history, previous affected children, and abnormal ultrasound findings. Participants underwent prenatal diagnostic tests, including chorionic villus sampling or amniocentesis, or fetal blood by various molecular techniques and enzyme-based studies. Data regarding patient demographics, types of disorders screened, and diagnostic outcomes were collected and analyzed.

**Results:**

Of the 1738 cases, 467 (26.87%) prenatal samples were identified as being affected by genetic anomalies. The diagnosed conditions included hematological disorders (*n* = 735/1738, 42.28%), inborn errors in metabolism (*n* = 513/1738, 29.52%), neurological disorders (*n* = 310/1738, 17.84%), musculoskeletal disorders (*n* = 45/1738, 2.59%), and other rare genetic disorders (*n* = 135/1738, 7.77%). Early diagnosis facilitated timely medical information and provided options for prevention, such as medical termination of pregnancy (MTP) in affected cases after genetic counseling.

**Conclusion:**

Our study demonstrates that prenatal diagnosis for rare genetic disorders is an invaluable step toward reducing the burden of these conditions. The use of advanced genetic techniques, combined with genetic counseling, enables effective prevention strategies. However, challenges such as accessibility, cost, and ethical considerations continue to pose barriers to widespread implementation in India. Increased awareness and government policy support are essential to make these diagnostic services universally available and affordable.

**Supplementary Information:**

The online version contains supplementary material available at 10.1186/s13023-025-04003-9.

## Background

The ability to identify ∼ 7,000 rare genetic disorders (RGD) has been made possible by advances in more recent genomic technology. However, the vast majority of these diseases are either untreatable or too expensive to treat. Prenatal diagnosis (PND) coupled with genetic counselling is therefore the only way to reduce the burden of several common and rare genetic disorders in many developing nations, including India.

Rare genetic disorders represent a broad category that includes a variety of conditions, each defined by distinct genetic variation or abnormalities [1]. Despite their individual rarity, the cumulative impact of these diseases affects a significant percentage of the global population, with an estimated 1 in 14 individuals suffering from a rare disorder [2]. According to estimates from the World Health Organization (WHO), there are about 7,000 known rare diseases that affect over 300 million individuals globally [3]. Over 80% of these diseases have a hereditary or genetic component [4]. Approximately 70% of these conditions develop in children, and 95% of them do not have Food and Drug Administration (FDA) approved therapies [5]. The complex genomic foundation, clinical pleiotropy, and paucity of therapy choices for several diseases frequently make them difficult to treat [6]. Additionally, there are still constraints to overcome, including inadequate access to advanced diagnostic equipment in rural areas where 60% of the Indian population resides, discrepancies in the resources available for healthcare, and the necessity for multidisciplinary cooperation for productive integration of research outcome into clinical practice [7].

Rare genetic disorders in India represent distinctive genetic diversity and local demographics, along with the broad worldwide trends [8]. With a population of more than 1.4 billion, India has a substantial number of patients with RGD. This number is impacted by factors such as consanguinity, socio-economic disparities, geographical location, and cultural practices [9]. Collaborative efforts between academic institutions, research centers, and healthcare providers have led to the advancements in genetic testing technologies, genetic counseling services, and the establishment of registries for rare diseases [10]. These initiatives aim to enhance diagnostic accuracy, improve patient outcomes, and inform public health policies related to rare genetic disorders [11].

Approximately 6–7% of all newborns in India, or 1.4 million annually, are born with serious birth defects, with over 50% of the cases without a known etiology [12]. Globally, India accounts for nearly 21% of the global birth defect mortality in the early neonatal period [13]. Recognizing the immense impact of rare genetic disorders and birth defects on the country’s public health landscape, genetic counselling and PND are the cornerstones of primary preventive management [14–18]. Pioneering studies have explored the application of genetic testing technologies such as next-generation sequencing (NGS), microarray analysis, and targeted gene panels in prenatal testing [19].

Prenatal diagnosis of neural tube defects (NTDs), hemoglobinopathy disorders (β-thalassemia, sickle cell anemia, and hemophilia), and lysosomal storage disorders have witnessed significant advancements in recent years, offering implications for preventive healthcare strategies worldwide. Early detection facilitates timely medical intervention, informed decision-making, and psychological preparation for parents [16, 19–24]. As technology continues to advance, the role of prenatal diagnosis (PND) and genetic counseling will become integral to prenatal care, ultimately improving outcomes for both parents and their children [25].

The present study aims to underscore the importance of early detection and intervention in mitigating the impact of rare genetic disorders on individuals, families, and societies. This study provides a 14-year retrospective overview of PND for different genetic disorders carried out at a tertiary genetic center in India.

## Materials and methods

### Patient cohort details

This retrospective study was carried out from 2008 to 2022 at the FRIGE’s Institute of Human Genetics, a tertiary referral center specializing in genetic disorders. Prenatal testing was performed if there was a parental or family history of any of the following conditions: β-thalassemia, sickle cell disease, hemophilia, DMD, SMA, a previous pregnancy with a known genetic disorder, a significant obstetric history, or ultrasound sonography (USG) findings such as increased nuchal translucency (NT), non-immune hydrops, or skeletal malformations indicating fetal abnormalities. Each couple was provided with a pre-test genetic counselling, which included review of prior obstetric outcomes, and explanation for indication of prenatal testing. Information regarding the suspected disorder, inheritance pattern, recurrence risk, and available diagnostic options (such as chorionic villus sampling, amniocentesis, and fetal imaging) was shared in clear, non-technical language. The potential outcomes of testing, limitations of the techniques, and implications for the pregnancy were also discussed. Informed consent was obtained from the parents in accordance with the principles outlined in the 1975 Helsinki Declaration. The study received approval from the institutional ethics committee. The present study included a total of 1,738 prenatal samples (cases), comprising amniotic fluid (AF) (*n* = 682), chorionic villus sample (CVS) samples (*n* = 1,052), and fetal blood (*n* = 4).

Prenatal testing was performed using one of the invasive procedures, specifically amniocentesis or chorionic villi sampling (CV sampling). CV sampling was carried out by obtaining a placental sample either by trans-cervical or trans-abdominal route, guided by USG, during the gestation period of 11 to 13 weeks. Amniocentesis involved aspiration of amniotic fluid (AF) through the abdominal wall using a hollow needle, also guided by ultrasonography, typically performed around 15 to 16 weeks of gestation. Genomic DNA was extracted from direct AF or CV sample or using cultured CV or AF cells by the salting-out method, quantified using QIAxpert (Qiagen, Hilden, Germany), and stored at -20 °C for subsequent analysis [26]. In instances where inborn metabolic abnormalities, particularly lysosomal storage disorders (LSDs), were suspected, AF or CV cells were cultured using special fibroblast culture media as per the protocol described previously [27, 28] and subjected to biochemical examination where molecular study was not available or not done in index case.

### Molecular genetic testing methodologies for common genetic disorders

**Fig. 1:**
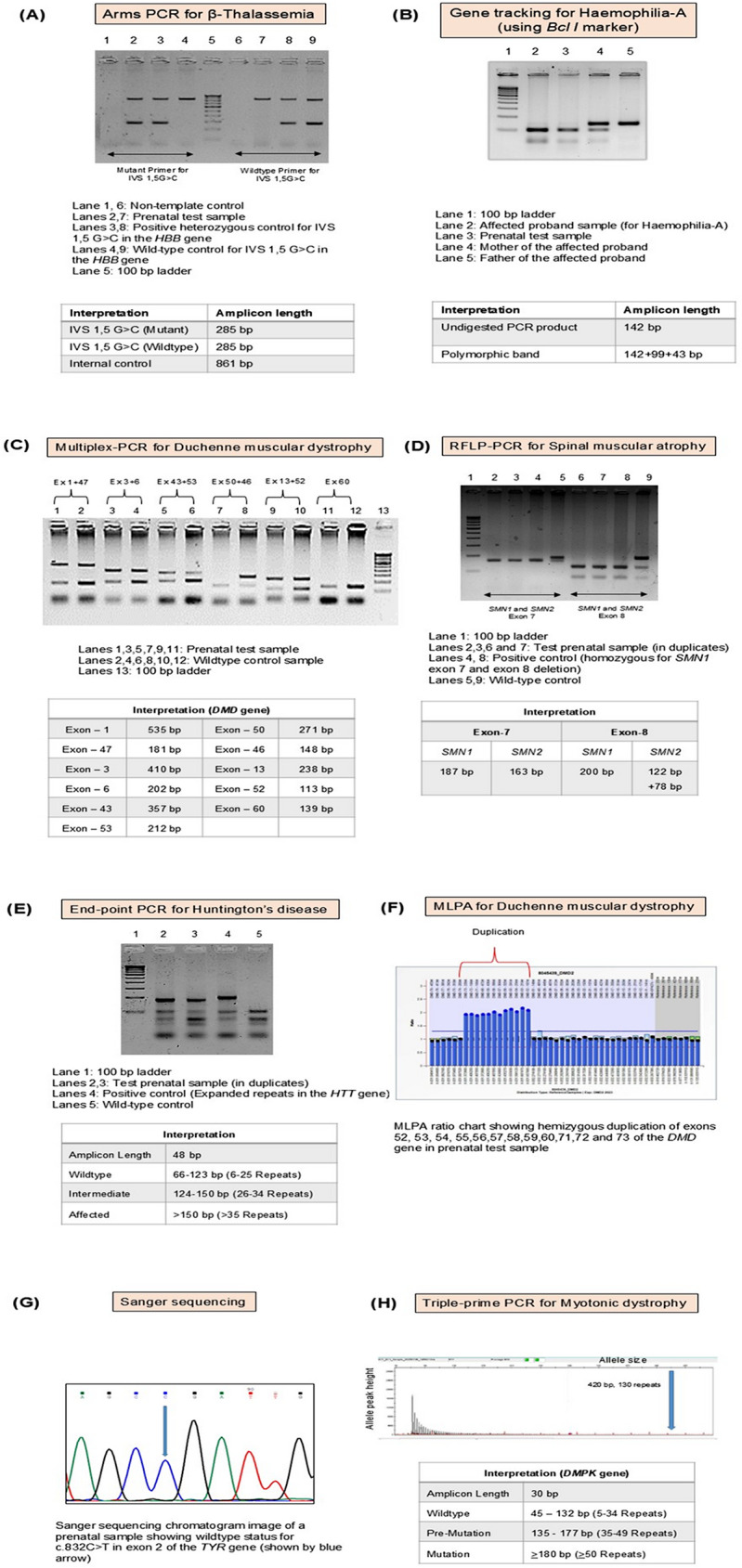
Representative laboratory findings of different genetic tests used for prenatal genetic diagnosis in the present study. (**A**) Gel image of ARMS-PCR for β-thalassemia. Prenatal test sample showing amplification with mutant primer only (band at 285 bp). Thus, confirming homozygous status for the variant IVS 1,5G > C in the *HBB* gene (affected). (**B**) Gel image of gene tracking for hemophilia A. Prenatal test sample showing band pattern same as that of the affected proband post restriction digestion by *Bcl*I. Thus, confirming the presence of the affected allele in the fetus. (**C**) Gel image of multiplex-PCR for Duchenne muscular dystrophy (DMD). Prenatal test sample showing absence of band for exon 50 of the *DMD* gene. Thus, confirming hemizygous deletion of exon 50 of the *DMD* gene (affected). (**D**) Gel image of PCR-RFLP for spinal muscular atrophy. Prenatal test sample showing absence of bands 187 bp and 200 bp for exon 7 and exon 8 of the *SMN1* gene, respectively. Thus, confirming homozygous deletion of exons 7 and 8 of the *SMN1* gene in the fetus (affected). (**E**) Gel image of end-point PCR for Huntington’s disease. Prenatal test sample showing band at ∼ 190 bp, i.e., ∼ 47 repeats. Thus, confirming the presence of the expanded CAG repeats in the *HTT* gene (affected). (**F**) MLPA probe ratio chart for Duchenne muscular dystrophy (DMD). Prenatal test sample showing a ratio of ∼ 1.8-2.0 for exons 52–60 and 71–73. Thus, confirming hemizygous duplication of exons 52–73 of the *DMD* gene in the fetus (affected). (**G**) Sanger chromatogram image of the prenatal test sample for the *TYR* gene, exon 2, showing homozygous wild-type status for the variant c.832 C > T in the *TYR* gene. Thus, confirming the fetus is unaffected by albinism. (**H**) Electropherogram results of triple-prime PCR for myotonic dystrophy. Prenatal test sample showing expanded allele with 130 CTG repeats in the *DMPK1* gene (affected)

Several genetic testing methodologies were employed in the study to conduct prenatal diagnoses of common genetic disorders. All genetic testing was carried out at FRIGE’s Institute of Human Genetics, Ahmedabad. The methods used, along with their application, advantages, and disadvantages are summarized in Table 1; Fig. 1. The selection of the testing strategy was guided by clinical history, family history and availability of prior genetic information. A detailed description of the genes and the specific variants tested using the listed methodologies, along with the primer sequences used in Sanger sequencing is provided in Additional file 1.

**Table 1:** Overview of the methodology used for prenatal genetic diagnosis in the present study

Test name	Advantages	Disadvantages	Disease name (OMIM)	Reference
Amplification refractory mutation system- Polymerase Chain Reaction (ARMS-PCR)	• Highly specific for known point mutations • Cost-effective • Quick turnaround time	• Not suitable for unknown or novel variants • Limited multiplexing	Cystic fibrosis (#219700)	[29]
			β-Thalassemia (#613985)	[30]
Gene-tracking using restriction fragment length polymorphism sites	• Cost-effective • Suitable where causative variant is unknown	• Interpretation can be complex • Expensive	Hemophilia-A (#306700)	[31]
Sanger sequencing	• High accuracy • Gold standard for known variant confirmation • Can detect novel variants	• Low throughput • Not suitable for large genes • Expensive per base compared to NGS	Cystic fibrosis (#219700)	[32]
			Congenital Adrenal Hyperplasia (#201910)	[32]
			Albinism/Oculocutaneous Albinism type 1 A (#203100)	[32]
			Crigler-Najjar syndrome (#606785)	[32]
			β-Thalassemia (#613985)	[32]
Multiplex-polymerase chain reaction (Multiplex-PCR)	• Simultaneous amplification of multiple targets • Cost-effective • Suitable for screening large genes with common deletions	• Primer design is complex • Risk of non-specific amplification or primer-dimer formation	Duchenne muscular dystrophy (#310200)	[33, 34]
			α-Thalassemia (#604131)	[35]
Restriction fragment length polymorphism-polymerase chain reaction (RFLP-PCR)	• Useful for common variants	• Expensive • Interpretation can be complex in some cases	Spinal muscular atrophy (#253300)	[36]
			Achondroplasia (#100800)	[37]
Multiplex ligation probe amplification (MLPA)	• Detects deletion/duplication at a single exon level • High-throughput	• Cannot detect point variants • Cannot map exact breakpoint of the deletion/duplication	Spinal muscular atrophy (#253300)	[38]
			Duchenne muscular dystrophy (#310200)	[39]
Triplet prime PCR	• Effective for detecting repeat expansion disorders	• Provides an approximation of the repeat count	Myotonic dystrophy (#160900)	[40]
			Friedreich ataxia (#229300)	[41]
End-point PCR	• Simple • Low-cost	• Provides an approximation of the repeat count	Huntington’s disease (#143100)	[42]

### Clinical exome sequencing/whole exome sequencing for rare diseases

Clinical exome sequencing (CES) was carried out after 2015 with the intervention of NGS, in instances where USG indicated a clinical suspicion of a genetic disease due to phenotypes correlating with neurological, musculoskeletal, or IEM disorders, whilst excluding cases with prior history of monogenic disorders within the family. In contrast, whole exome sequencing (WES) was utilized for cases where there were multiple abnormal USG findings without any specific differential diagnosis. CES capture was performed using a custom capture kit and the sequencing was carried out on Illumina HiSeq 2500 platform (Illumina, California, USA) at an average coverage of 100x while WES capture was performed using Agilent SureSelect V6 enrichment kit (Agilent, California, USA) or Twist Human Core Exome kit v2.0 (Twist Biosciences, California, USA). The prepared library was subjected to sequencing with a mean coverage of ∼ 100x on the Illumina HiSeq 2500 or NovaSeq 6000 platform (Illumina, USA). Sequences obtained as FASTQ files were aligned to the human reference genome (GRCh37/GRCh38) using BWA MEM v0.7.12 [43]. Single nucleotide variations (SNVs) and indels were called using GATK v4.12 Haplotype caller [44]. In addition to SNVs and small indels, copy number variants (CNVs) were detected from the data using the ExomeDepth v1.1.10. The variant annotation, filtration and prioritization protocol was executed using the previously established methodology [45].

### Enzyme assay for prenatal diagnosis of lysosomal storage disorders (LSDs)

Prenatal testing for LSDs was carried out by either molecular or enzymatic methods. Enzyme testing was carried out in CV, cultured CV or cultured AF (CAF) cells based on the prior clinical history or enzyme test report in the previously affected child. Enzyme assay was performed by standard protocol for a given enzyme using 4-methyl umbelliferyl (MU) fluorometric assay or para-nitrocatechol sulfate (PNCS) spectrophotometric synthetic substrate as outlined previously [46, 47]. Enzyme and molecular analysis was carried out on CV cells in 177 and 61 cases, while CAF cells were analyzed in 251 and 23, respectively. 1 fetal blood sample was tested by molecular technique using a method described previously [46]. The enzyme activity was expressed as nmol/h/mg of protein, except for nmol/4 h/mg of protein for α-iduronate sulfatase and nmol/17 h/mg of protein for β-galactose-6-sulfate-sulfatase, heparan sulfamidase, and β-galactocerebrosidase. Prenatal testing included simultaneous testing of prenatal samples with positive and negative controls for the specific enzymes tested.

## Results

### Patient cohort

**Fig. 2:**
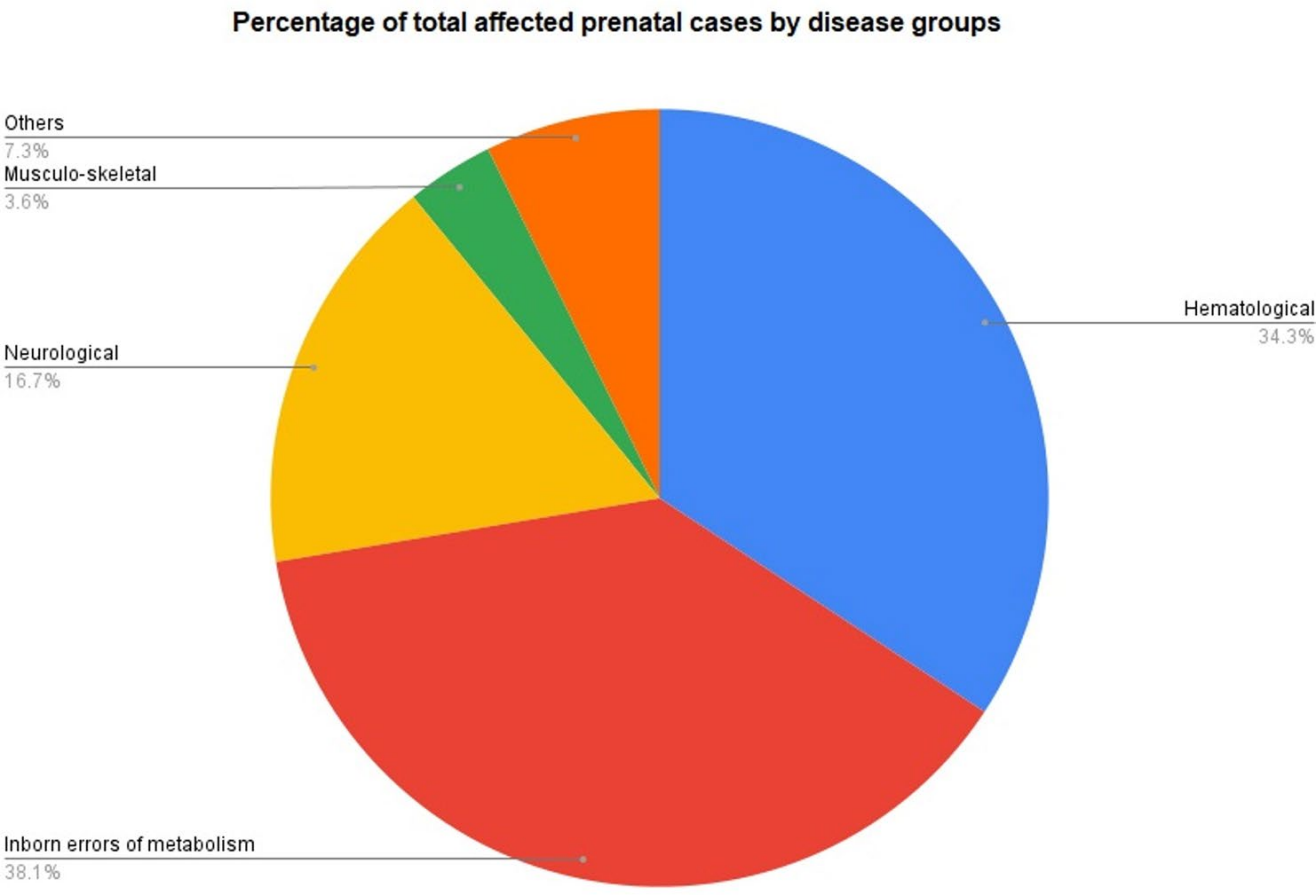
Percentage of total affected prenatal cases by disease group

Our center provided prenatal diagnosis (PND) to 1,738 couples over a 14-year period from 2008 to 2022. During this period prenatal testing was performed on 1052 (60.52%) CVS, 682 (39.24%) AF, and 4 (0.23%) fetal blood samples (Table 2). Of 1,738 cases, PND for hematological disorders was offered in 735 (42.04%) cases, for inborn error of metabolism (IEM) in 513 (29.51%) cases, for neurological disorders in 310 (17.83%) cases, for musculoskeletal disorders in 45 (2.58%) cases, and for other rare genetic disorders in 135 (7.76%) cases. Figure 2 provides the distribution of total affected prenatal cases under the 5 main disease groups. The other rare genetic disorder groups for which PND was offered included deafness (*n* = 10), dermatological (*n* = 31), endocrine (*n* = 24), hepatic (*n* = 4), mitochondrial (*n* = 8), nephrology (*n* = 10), ophthalmic (*n* = 9), pulmonary (*n* = 32), immunology (*n* = 5), and cardiology sub-group (*n* = 2).

**Table 2:** Demographics of cases recruited

Characteristics	Total cases (*N* = 1738)
**Total cases**, ***n*** **(%)**	
Normal	752 (43.27%)
Carrier	519 (29.86%)
Affected	467 (26.87%)
**Sample**, ***n*** **(%)**	
CVS	1052 (60.52%)
AF	682 (39.24%)
Fetal blood	4 (0.23%)
**Consanguinity**, ***n*** **(%)**	
Consanguineous	160 (9.21%)
Non-consanguineous	1307 (75.20%)
No data	271 (15.60%)
**Clinical history**, ***n*** **(%)**	
Previous affected child or family history	1698 (97.69%)
USG findings	40 (2.30%)

CVS = chorionic villus sampling, AF = amniotic fluid sampling

We observed consanguinity in 9.2% (*n* = 160/1738) of the total cases whilst in 75.2% of cases (*n* = 1307/1738), there was no reported consanguinity. In the remaining 15.6% of cases (*n* = 371/1738), consanguinity status was unknown due to the lack of clinical or ethnic origin history. PND was offered due to a prior history of a known genetic disorder in 97.6% (*n* = 1698/1738) of cases. In 2.3% of cases (*n* = 40/1738) fetal anomalies were detected by USG screening and hence they were referred for PND.

**Fig. 3:**
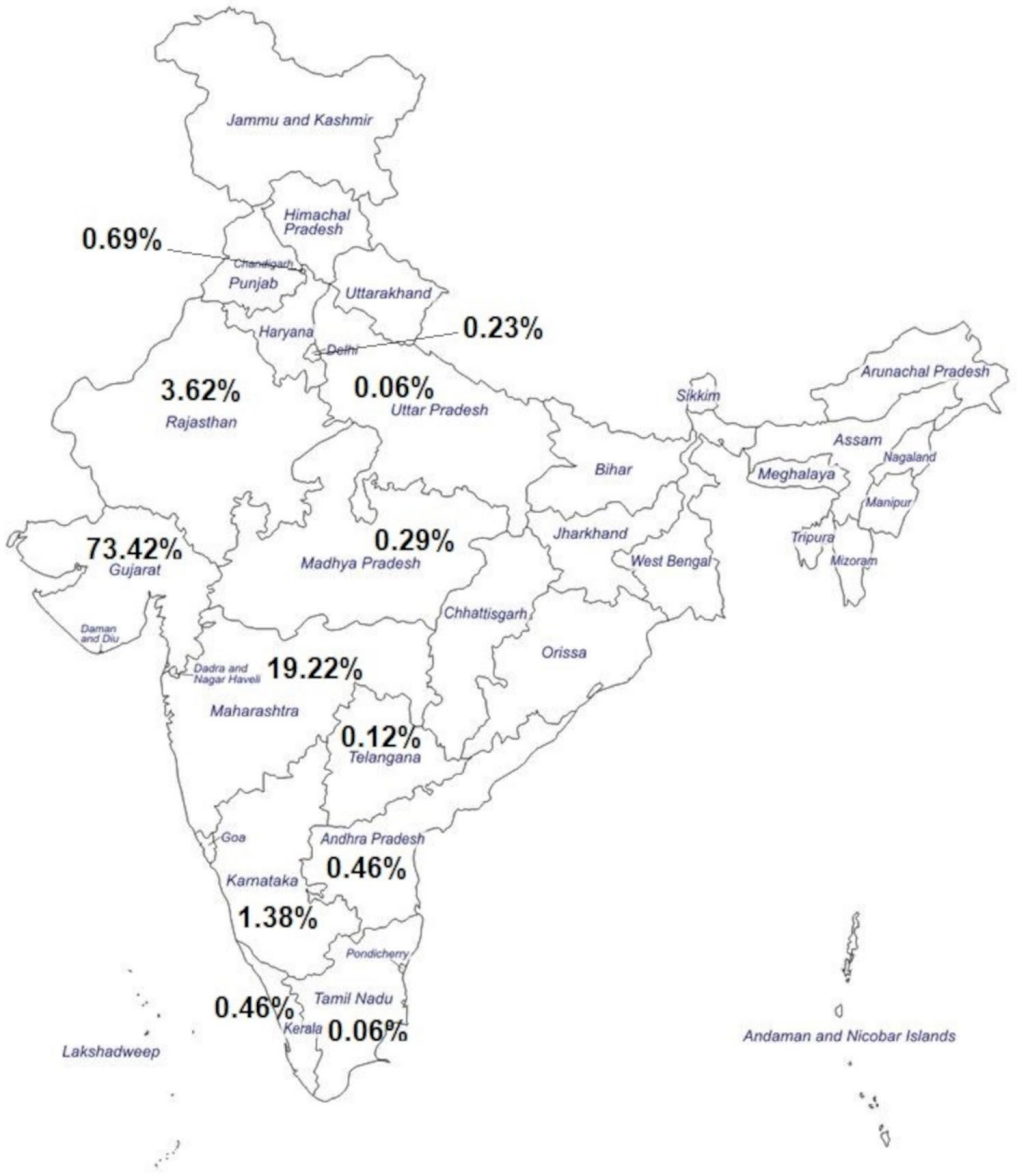
Geographical distribution of total cases received for prenatal diagnosis in the present study

The highest number of cases for PND were referred from Gujarat (*n* = 1276/1738; 73.42%), followed by Maharashtra (*n* = 334/1738; 19.22%), Rajasthan (*n* = 63/1738; 3.62%), Karnataka (*n* = 24/1738; 1.38%) and Chandigarh (*n* = 12/1738; 0.69%). Figure 3 provides distribution of total cases referred for PND from across 12 states in India. Additional file 2 provides a detailed state-wise distribution of the total number of cases referred for PND across all genetic disorder groups.

### Hematological group

Of the 1,738 prenatal samples tested, 735 cases (42.28%, *n* = 735/1738) were referred for PND of hematological disorders. In total, consanguinity was found in 3.67% cases (*n* = 27/735), whereas in 86.66% cases (*n* = 637/735) there was no consanguinity. In 9.66% (*n* = 71/735) of the cases, consanguinity status was unavailable due to insufficient data. In 93.74% of cases (*n* = 689/735) in this group, there was a previous history of an affected child with one of the hematological disorders. In 6.26% of cases (*n* = 46/735) report of biochemical studies in parents was available which was suggestive of a hematological disorder. Of the 735 prenatal samples tested, 521 were CV samples and the remaining 214 were AF samples. Prenatal testing for β-thalassemia was carried out in 657 samples, sickle cell anemia in 44 samples, hemophilia in 27 samples, and in 7 cases for other hematological disorders. Of these, 146 cases (22.22%, *n* = 146/657) were confirmed to be affected with β-thalassemia, 6 cases (13.64%, *n* = 6/44) were affected with sickle cell anemia, 6 cases (22.22%, *n* = 6/27) with hemophilia-A, and one case each (28.57%, *n* = 2/7) with α-thalassemia and Fanconi anemia. Telephonic follow-up of all affected cases had confirmed that parents opted for MTP after genetic counselling.

### Inborn errors of metabolism group

Prenatal testing was offered for IEM disorders in 513 cases (29.52%, *n* = 513/1738). Of these cases, 96.9% (*n* = 497/513) were referred for testing due to a clinical history of a previous child affected with a known IEM disorder, while in 3.12% of cases (*n* = 16/513), USG findings were suggestive of fetal hydrops. Overall, sample type distribution was as follows: 238 CV, 274 AF, and 1 fetal blood sample. Family pedigree details identified 17.93% (*n* = 92/513) of the cases with consanguinity in this group. In 58.47% of cases (*n* = 300/513), there was no consanguinity, and data regarding consanguinity was not available for 23.59% of cases (*n* = 121/513). Among the 513 cases, 51 cases (9.94%, *n* = 51/513) were tested for small molecule IEM disorders (e.g., organic acidemia, amino acid disorders, and fatty acid defects), and 462 cases (90.06%, *n* = 462/513) were tested for large molecule IEM disorders (e.g., LSDs). In the small molecule IEM disorder group, a total of 11 prenatal cases (21.57%, *n* = 11/51) tested positive. These included 2 cases (18.2%, *n* = 2/11) of methylmalonic encephalopathy followed by 1 case (9.1%, *n* = 1/11) each of carnitine acylcarnitine translocase deficiency, Hartnup disorder, HMG-CoA lyase deficiency, HMG-CoA synthase-2 deficiency, maple syrup urine disease type IA, methylmalonic aciduria, methylmalonic aciduria type mut (0), ornithine transcarbamylase deficiency, and tyrosinemia type 1, respectively (Additional file 3). Of the 462 prenatal cases tested under the large molecule IEM subgroup, molecular study was carried out in 34 cases (7.36%, *n* = 34/462) where variant details were known in the index case along with a confirmed parental carrier status for the identified variant. Enzyme study was carried out in 428 cases (92.64%, *n* = 428/462) where genetic study was not performed in the index case. Overall, we observed 167 cases (36.15%, *n* = 167/462) confirmed to be affected with 23 different disorders. The highest number of affected cases in this group belonged to Hunter disease (MPS-II) (16.17%, *n* = 27/167), followed by Krabbe disease (11.98%, *n* = 20/167), and GM-1 gangliosidosis (10.18%, *n* = 17/167) (Additional file 3).

### Neurological disorders

Prenatal testing was offered for 310 cases (17.84%, *n* = 310/1738) under the neurological group. Among these, 97.7% of cases (*n* = 303/310) were referred due to a clinical history of a previously affected child. In 7.69% (*n* = 7/310), USG findings suggested one or more than one of the following observations: hydrocephalus, macrocephaly, prominent lateral ventricle including fronto-temporal space, perifocal edema, chronic hemorrhage, mild facial hypoplasia, short nose, flat nasal bridge, postaxial clinodactyly in all fingers, premature ossification of coccyx, premature calcification, absence of nasal bone, and fetal akinesia deformity syndrome in the fetus. The sample type distribution for PND in this group was as follows: 200 CV samples, 109 AF samples, and one fetal blood sample. Consanguinity was observed in 6.45% of cases (*n* = 20/310), whereas in 79.35% of cases (*n* = 246/310) there was non-consanguinity. Data regarding consanguinity was not available for 14.19% of cases (*n* = 44/310). Overall, the highest number of prenatal cases were tested for SMA (*n* = 110/310) and DMD (*n* = 101/310), followed by other rare disorders (*n* = 99/310). We observed 25.16% of cases (*n* = 78/310) tested positive for one of the 22 different neurological disorders (Additional file 3). Of these, the majority were of SMA (37.18%, *n* = 29/78), followed by DMD (29.49%, *n* = 23/78).

### Musculoskeletal group

Prenatal testing was performed in 45 cases (2.59%, *n* = 45/1738) due to suspicion of a musculoskeletal disorder. Among these, 73.33% of cases (*n* = 33/45) were referred due to clinical history of a previously affected child. In 26.66% of cases (*n* = 12/45), USG findings showed one or more of the following observations: polyhydramnios, shortening of femur, clubfoot, generalized micromelia, bowing of long bones, small thorax with protuberant abdomen, lethal skeletal dysplasia, skeletal dysplasia non-lethal variant short long bone, depressed nasal bridge, dolichocephaly, dilated lateral and third ventricle, and oligohydramnios in the fetus. In this group, PND was conducted using 19 CV samples, 25 AF samples, and one fetal blood sample. Consanguinity was observed in 15.55% of cases (*n* = 7/45), whereas 64.44% of cases (*n* = 29/45) were non-consanguineous. Data regarding consanguinity was not available for 20% (*n* = 9/45) of the cases. Overall, 17 cases tested positive, each affected with a unique musculoskeletal disorder, details of which are provided in Additional file 3.

### Others rare disorder group

Prenatal testing in this group was conducted on 135 samples and the sample type distribution was, 74 CV samples, 60 AF samples, and one fetal blood sample. In 96.3% of cases (*n* = 130/135), there was a history of an affected child in the family, whereas in 3.7% of cases (*n* = 5/135), USG was suggestive of congenital hypothyroidism with goiter. The overall consanguinity was 10.37% (*n* = 14/135); non-consanguinity was observed in 70.37% of cases (*n* = 95/135), and in 19.26% of cases (*n* = 26/135), no consanguinity data was available. Overall, 34 cases tested positive among the ten rare genetic disorder sub-groups, details of which are listed in Additional files 3 and 4. The highest number of affected cases were found in the endocrine sub-group (32.3%, *n* = 11/34). We observed majority of the affected cases with congenital adrenal hyperplasia (26.4%, *n* = 9/34) in the endocrine sub-group and cystic fibrosis (17.6%, *n* = 6/45) in the pulmonary sub-group.

### Pattern of inheritance and pathogenicity in cases with confirmed diagnosis

Out of 1738 prenatal cases studied, 467 (27%) cases were diagnosed with a genetic disorder. The distribution of affected pregnancies based on the mode of inheritance of the genetic disorder was as follows: 406 (86.93%) prenatal cases were diagnosed with an autosomal recessive (AR) disorder, 21 (4.50%) prenatal cases exhibited an autosomal dominant (AD) mode of inheritance, and 40 (8.57%) prenatal cases presented with X-linked inheritance pattern. Of the affected fetuses with an AR disorder, 170 (41.87%) showed the presence of a homozygous variant, 85 (20.93%) harbored compound heterozygous variants, and in five cases (1.23%) a single heterozygous variant was identified. Importantly, 90.48% (*n* = 285/315) of affected prenatal cases showed presence of pathogenic or likely pathogenic variants, whereas in 9.52% (*n* = 30/315) of cases, variants of uncertain significance (VUS) were identified. In 25 cases, VUS were further reclassified as likely pathogenic after parental segregation analyses, whereas in 5 instances, the diagnosis remained inconclusive due to the identification of a single heterozygous variant in a clinically relevant gene associated with an autosomal recessive (AR) condition. It is important to note that the potential presence of a second disease-causing variant located in deep intronic or promoter regions, as well as the possibility of complex structural variants, cannot be excluded in these cases. Therefore, genetic counseling was provided to these families regarding continuation of pregnancy, but a follow-up study was not carried out about the outcome. Additional file 4 describes variant and gene details for all diagnosed prenatal cases in the present study. For all fetuses with a confirmed diagnosis for a genetic disorder, the clinical management involved a multidisciplinary approach. The core team included fetal medicine experts, clinical geneticists, neonatologists, and genetic counselors. The potential natural course of the disease, available treatment options, associated costs, supportive care options in cases where treatment is not available, and existing government schemes were discussed in detail with the family.

## Discussion

One of the available strategies for preventing the burden of genetic disorders is PND. The aim of the present study was to delineate the distribution of genetic disorders for which PND is commonly utilized in India and to raise awareness of disease prevention using PND. The utilization of various fetal tissues, such as chorionic villi sample, amniotic fluid, and fetal blood, for enzyme-based and/or molecular-based PND of various genetic abnormalities is highlighted in the study.

In countries like Sub-Saharan Africa, Middle East, Caribbean, and certain regions of the United States and Europe, PND has become an integral component of public healthcare strategies [48–50]. Likewise, nations with significant incidence of sickle cell anaemia, namely Ghana, Nigeria, and Saudi Arabia, are actively working to incorporate prenatal screening into their healthcare systems [51, 52]. India has a high carrier frequency for β-thalassemia and spinal muscular atrophy [53, 54], which explains the observation of the majority of affected prenatal cases with β-thalassemia (36%, *n* = 146/467), followed by spinal muscular atrophy (6%, *n* = 29/467) in the present study. A remarkable success in identifying β-thalassemia through national carrier screening programs, genetic counselling, and free or subsidized PND has been documented previously [55–58]. Italy and other Mediterranean countries have also integrated β-thalassemia prevention into their public health systems, providing widespread screening, prenatal testing, and counselling services [59, 60].

The present study recorded the highest referrals for PND from urban areas, especially from Gujarat. One of the reasons being FRIGE Institute of Human Genetics being located geographically within Gujarat, thereby, making it easier for patients from Gujarat to travel for pre- and post-test genetic counselling, and PND. India, while advancing in urban areas, faces challenges in expanding these services to rural populations and ensuring affordability for economically disadvantaged groups [61, 62]. To overcome this challenge, the Department of Biotechnology (DBT) has established Nidan Kendra in various districts across the country for easy access to testing centres. There is an increased awareness of carrier testing and this can be attributed as one of the reasons for the rising trend of PND for β-thalassemia. This observation is supported by a decrease in the number of births of children with β-thalassemia major in a recent study [45]. Surprisingly, prenatal cases with sickle cell anaemia diagnosed in the present study were less than expected, given the fact that an estimated 1–3% of the population in India is affected with sickle cell anaemia. Scheduled tribes and specific tribal communities in states such as Gujarat, Madhya Pradesh, Maharashtra, Chhattisgarh, and Odisha have a high prevalence of sickle cell anaemia [63–67]. Low accessibility to PND in these areas and less awareness as compared to β-thalassemia are likely to be the contributing factors. Unlike β-thalassemia, public awareness regarding DMD and SMA is still in its nascent stages in India, and hence literature data also suggests that the uptake of PND for SMA and DMD is low in the country [68]. The Rashtriya Bal Swasthya Karyakram (RBSK), a National Health Mission program, offers screening for genetic disorders and birth defects; however, the focus on SMA and DMD is minimal [69]. Expanding this program to incorporate SMA and DMD carrier screening and PND will significantly aid in early detection and prevention [70].

Of note, in 4.2% (*n* = 73/1738) of the cases under this group, PND was offered using an NGS-based sequencing study. Recently, several studies have assessed the utility of exome sequencing for PND, and the diagnostic yield ranged from 6.2 to 80% [71–73]. WES and CES have also become pivotal in diagnosing complex and rare musculoskeletal disorders [74, 75]. These tests allow precise detection of variants associated with disorders like osteogenesis imperfecta and skeletal dysplasia [76, 77]. Diagnostic yields are highest in cases of nonimmune fetal hydrops, skeletal dysplasias, and central nervous system anomalies [70]. The present study also documented a 77% diagnostic yield by WES and a 33% diagnostic yield by CES.

Fetal ultrasound is a fundamental tool for detecting structural abnormalities in the skeletal system. High-resolution and 3D ultrasound provide detailed images of fetal limbs, spine, and joints, allowing early diagnosis of conditions like clubfoot, arthrogryposis, and short-limb dwarfism [78, 79]. Developed nations like the US, the United Kingdom (UK), and Germany have standardized the use of high-resolution ultrasounds in routine prenatal care. Ultrasound is widely used to detect arthrogryposis, a condition characterized by joint contractures that can be observed as early as the second trimester [80, 81]. In India, while fetal ultrasound is routinely performed in both public and private sectors, the availability of advanced imaging technologies such as 3D/4D ultrasound and the expertise to interpret complex musculoskeletal anomalies are largely confined to urban tertiary care centres. Despite these challenges, the integration of prenatal ultrasound with emerging genetic testing methods is gradually improving the early diagnosis of musculoskeletal disorders. Globally, PND of musculoskeletal disorders has advanced with the advent of sophisticated genetic testing, non-invasive screening, and advanced imaging technologies [82, 83]. Countries like the US, the UK, and Germany lead in these best practices, ensuring widespread access to prenatal care, early detection, and multidisciplinary support for families [19, 84, 85].

Experience from our previous studies has shown the utility of prenatal samples, including CVS and AF, for enzyme-based diagnosis of LSDs. This was also observed in the present study, as we could offer enzyme-based PND in 25% of cases (*n* = 441/1738) where there was a history of an affected child with LSD in the family, USG abnormality showing fetal hydrops, or only biochemical study performed in the index case. Several studies have shown effective PND for LSDs using enzyme testing [21, 86, 87]. However, pseudo-deficiencies of a few lysosomal enzymes are frequently an issue with enzyme-based PND for specific LSDs [88]. In these situations, before providing PND, variant analysis and enzyme testing in the index and parents are required.

**Fig. 4:**
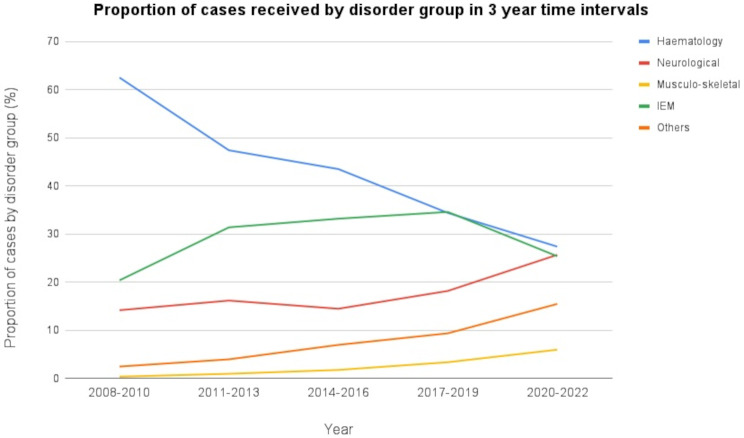
Proportion of cases received for prenatal diagnosis by disorder group in 3-year time intervals

Of note, the present study has shown a steady decline in the number of cases for PND in the hematological and IEM groups. This trend may be attributed to the increase in the number of government and private centres offering prenatal diagnostic services as well as preimplantation genetic diagnosis in many of the in vitro fertility (IVF) clinics. Conversely, the rise in cases for PND of neurological, musculoskeletal, and other rare genetic disorders suggests a growing awareness of these conditions, facilitated by advancements in technology (Fig. 4).

India has the highest infant mortality rate (IMR) at about 29.1 deaths per 1000 live births, according to the UNICEF [89]. Despite this, India currently does not have a national policy for newborn screening, although, several initiatives aimed at improving maternal health and lowering infant mortality have been established. For example, the Pradhan Mantri Surakshit Matritva Abhiyan (PMSMA) offers pregnant women free, guaranteed, and high-quality prenatal checks on the ninth of each month [90]. Furthermore, by implementing the National Policy for Rare Diseases (NPRD), in 2021, India has achieved notable progress in tackling rare genetic diseases [91]. This policy offers an organized method for diagnosing, treating, and managing rare diseases; providing financial help and improving healthcare infrastructure for those affected. Prenatal detection enables prompt delivery planning at specialized centres and enables access to early interventions like gene therapy, hematopoietic stem cell transplantation, or enzyme replacement therapy when a fetus is diagnosed with a treatable condition like SMA or MPS type I [92]. These interventions frequently lead to significantly improved survival and functional outcomes. Early detection of IEMs such as phenylketonuria or galactosemia allows for rapid implementation of postnatal dietary changes and reducing long term morbidity and mortality [93]. PND helps families make decisions about continuation or discontinuation of a pregnancy, based on the phenotypic expressivity & pleiotropy, availability of treatment, and long-term morbidity and mortality. Especially in several IEM disorders such as phenylketonuria, medium and very-long chain fatty acid oxidation disorders, and hypothyroidism, PND helps in preparing neonatal treatment plans to significantly reduce long term morbidity.

While there are several merits to the study, there are some limitations too: (a) All patients were provided with a post-test genetic counselling, however, follow-up on the outcome of the PND was not possible in all cases. Therefore, the overall impact of the PND on pregnancy couldn’t be elucidated in the present study. (b) In 5 cases, only a single heterozygous variant was detected in a gene associated with an autosomal recessive disorder. Despite extensive post-test genetic counselling, the parents were lost to follow-up and their pregnancy outcome couldn’t be recorded. (c) The present study only focussed on diseases with molecular or biochemical genetic aetiology. The study did not include cases with chromosomal abnormalities identified through PND.

## Conclusion

Overall, the current study suggests that prenatal diagnosis (PND) is essential in reducing the burden of genetic disorders and provides pregnant couples with important information regarding the health of their fetus. Techniques such as chorionic villus sampling and amniocentesis can enable early detection of genetic disorders. The most common genetic disorders for which PND is routinely being used includes sickle-cell anaemia, β-thalassemia, IEM disorders, neurological disorders such as SMA and DMD, as well as other uncommon genetic conditions like cystic fibrosis. Additionally, PND is employed when several USG anomalies are found. With an increase in the number of rare disease diagnoses, it will be important to pursue alternate disease management strategies. It is also imperative that the national and state government initiatives be implemented to raise awareness of targeted prenatal testing as a preventive measure against common genetic diseases in India, given the high cost of treatment and a growing demand for public healthcare resources in disease management.

## Supplementary Information

Below is the link to the electronic supplementary material.

Additional file 1: Details of the different test methodologies used for the testing of the genes and specific variants, along with primer sequences used for Sanger sequencing

Additional file 2: State-wise distribution of total cases in the present study based on their referral clinic

Additional file 3: Detailed distribution of total number of affected prenatal cases across five disease groups

Additional file 4: Details of genes and variants identified in total affected prenatal cases across five disease groups

## Data Availability

All data supporting the findings of this study are available within the paper and it’s supplementary information.

## Abbreviations

RGD: Rare genetic disorder
WHO: World health organization
FDA: Food and Drug Administration
NGS: Next generation sequencing
NTDs: Neural tube defects
PND: Prenatal diagnosis
USG: Ultrasound sonography
CVS: Chorionic villus sampling
AF: Amniotic fluid
CV: Chorionic villus
LSD: Lysosomal storage disorder
PCR: Polymerase chain reaction
ARMS-PCR: Amplification refractory mutation system- Polymerase chain reaction
CF: Cystic Fibrosis
DMD: Duchenne muscular dystrophy
MLPA: Multiple ligation probe amplification
MCA-PSV: Middle cerebral artery peak systolic velocity
RFLP: Restriction fragment length polymorphism
SMA: Spinal muscular atrophy
HD: Huntington’s disease
SCA: Spinocerebellar ataxia
FA: Friedreich’s ataxia
MD: Myotonic dystrophy
TP-PCR: Triple Primed PCR
CES: Clinical exome sequencing
WES: Whole exome sequencing
SNV: Single nucleotide variant
CNV: Copy number variant
CAF: Cultured amniotic fluid
MU: 4-methyl umbelliferyl
PNCS: Para-nitrocatechol sulfate
IEM: Inborn error of metabolism
AR: Autosomal recessive
AD: Autosomal dominant
VUS: Variants of uncertain significance
DBT: Department of biotechnology
NHM: National Health Mission
ACOG: American College of Obstetricians and Gynecologists
NMDs: Neurological disorders
MDA: Muscular Dystrophy Association
RBSK: Rashtriya Bal Swasthya Karyakram
PND: Prenatal diagnosis
ERT: Enzyme replacement therapies
CAH: Congenital adrenal hyperplasia
IMR: Infant mortality rate
NBS: Newborn screening
ICMR: Indian Council of Medical Research
21-OHD: 21-hydroxylase deficiency
